# The complete chloroplast genome sequence of *Prunus discadenia* (Rosaceae), a species with great ornamental value

**DOI:** 10.1080/23802359.2021.1888336

**Published:** 2021-03-16

**Authors:** Yu-Hui Guo

**Affiliations:** College of Life Sciences, Northwest A&F University, Yangling, Shaanxi, China

**Keywords:** *Prunus discadenia*, phylogenetic analysis, chloroplast genome, Rosaceae

## Abstract

*Prunus discadenia* is a *Cerasus* species with great ornamental value and endemic to China. Here, the complete chloroplast (cp) genome of *P. discadenia* was assembled based on the Illumina reads. The cp genome is 157,915 bp in length, which contains two inverted repeat regions (26,415 bp) separated by the small single copy (19,119 bp) and the large single copy (85,966 bp) regions. The plastome contains 131 genes, and the overall GC content is 36.7%. Complete chloroplast genome of *P. discadenia* is of great significance to rebuilt the phylogeny of *Cerasus*.

*Cerasus*, a subgenus of *Prunus* s.l. (Rosaaceae), is distributed widely in the Northern Hemisphere. With beautiful flowers and edible fruits, this group has been subjected to considerable scientific attention due to its horticultural and economic value. *Prunus discadenia* (Koehne) S. Y. Jiang & C. L. Li, a member of *Cerasus*, has great and special horticultural and economic value due to its subcorymbose-racemose inflorescence and the leave and stipule margin with capitate glands, which distinguishes it from other species significantly (Lu 1986). It is mostly distributed in Sichuan and Shaanxi Province of China. Here, we used genome skimming approach (Zimmer and Wen 2015) to get the complete chloroplast genome information of *P. discadenia* (GenBank accession number: MK905683).

The fresh leaves of *P. discadenia* were collected in Ningshan county, Shaanxi Province, China (108°29′30ʺE, 33°28′48ʺN, alt. 2242 m) and dried with silica gel. A voucher specimen (ZL201708002) was deposited in the Herbarium of Northwest A&F University (WUK), China. After extracting total genomic DNA with CTAB method (Doyle and Doyle 1987), a 500-bp DNA TruSeq Illumina (Illumina Inc., San Diego, CA, USA) sequencing library was constructed and then sequenced using Illumina Miseq platform (Illumina). Reads of the chloroplast genome were assembled with GetOrganelle (Jin et al. 2020). The complete cp genome was annotated with PGA (https://github.com/quxiaojian/PGA).

The complete cp sequence of *P. discadenia* is 157,915 bp long with 131 genes, including 86 protein-coding genes, 37 tRNA genes, and 8 rRNA genes. Most genes only have one copy, while six tRNA genes (*trnA-UGC*, *trnG-GCC*, *trnI-GAU*, *trnK-UUU*, *trnL-UAA*, and *trnV-UAC*), six protein-coding genes (*atpF*, *ndhA*, *ndhB*, *rpl2*, *rpoC1*, *rps12*) are duplicated. Two protein-coding genes (*clpP* and *ycf3*) have three copies. The overall G/C content of *P. discadenia* cp genome is 36.7%. In the chloroplast genome, the length of each inverted repeat (IR) is 26,415 bp, while the lengths of the large single-copy (LSC) and the short single-copy (SSC) are 85,966 bp and 19,119 bp, respectively.

To clarify the phylogenetic position of *P. discadenia*, totally 20 species of Rosaceae were used to construct phylogenetic tree with RAxML. *P. discadenia* and five other species (*Prunus maximowiczii*, *P. campanulata*, *P. pseudocerasus*, *P. cerasoides*, and *P. rufa*) compose a clade (Figure 1), which proved that *P. discadenia* is a member of *Cerasus*. Further phylogenomic study is needed to tackle the relationship within *Cerasu*s.

**Figure 1.**
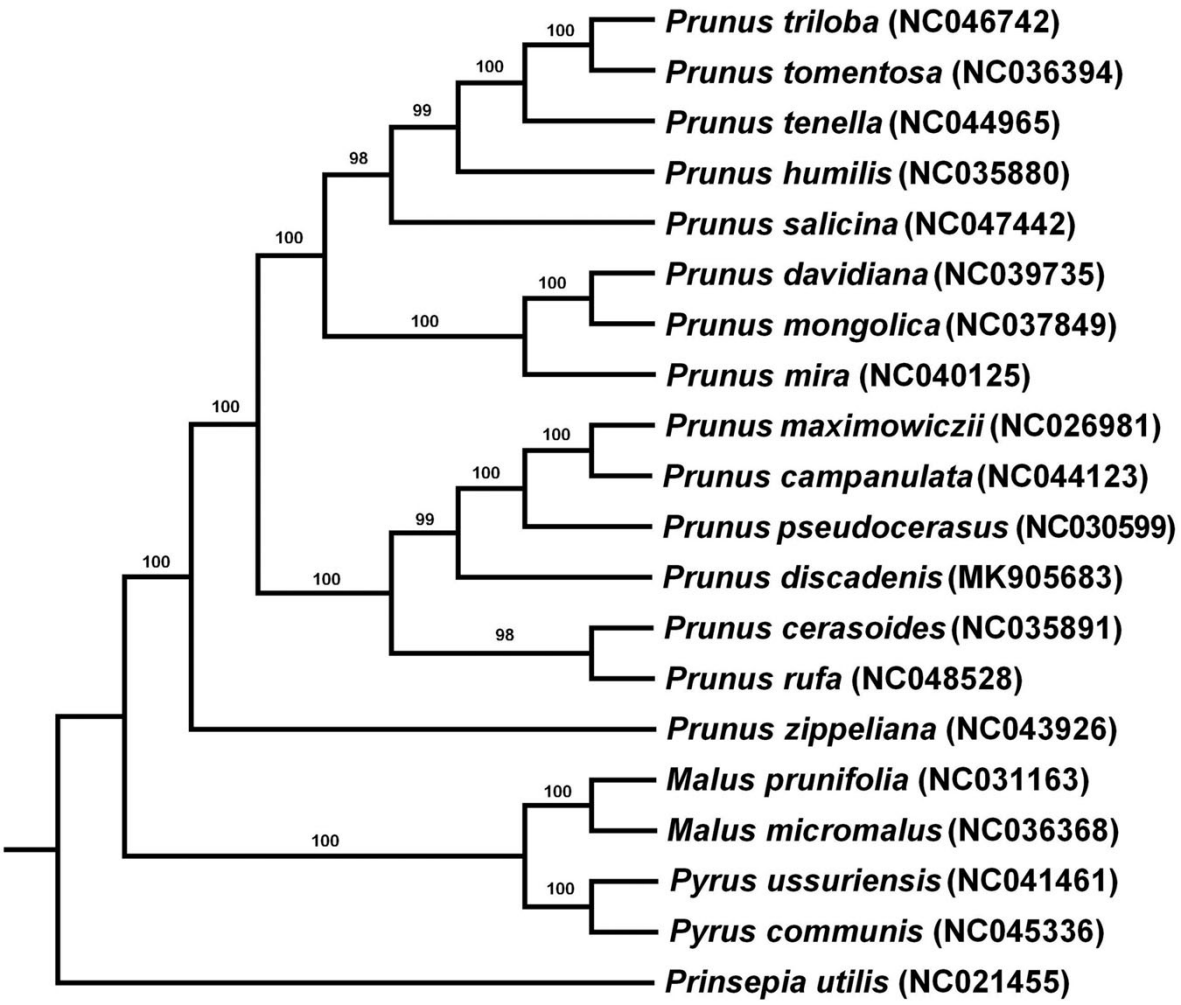
The maximum-likelihood tree based on the complete chloroplast genome of 20 species.

## Data Availability

The genome sequence data that support the findings of this study are openly available in GenBank of NCBI at [https://www.ncbi.nlm.nih.gov](https://www.ncbi.nlm.nih.gov/) under the accession no. MK905683. The associated BioProject, SRA, and Bio-Sample numbers are PRJNA671803, SRR12927899, and SAMN16552097 respectively.
